# Portable robots for upper-limb rehabilitation after stroke: a systematic review and meta-analysis

**DOI:** 10.1080/07853890.2024.2337735

**Published:** 2024-04-19

**Authors:** Kevin C. Tseng, Le Wang, Chunkai Hsieh, Alice M. Wong

**Affiliations:** a Department of Industrial Design, National Taipei University of Technology, Taipei, Taiwan, ROC; b Product Design and Development Laboratory, Taoyuan, Taiwan, ROC; c Department of Physical Medicine and Rehabilitation, Chang Gung Memorial Hospital at Taoyuan, Taoyuan, Taiwan, ROC

**Keywords:** Portability, stroke, robotics, exoskeleton, hand and upper extremity rehabilitation

## Abstract

**Background:**

Robot-assisted upper-limb rehabilitation has been studied for many years, with many randomised controlled trials (RCTs) investigating the effects of robotic-assisted training on affected limbs. The current trend directs towards end-effector devices. However, most studies have focused on the effectiveness of rehabilitation devices, but studies on device sizes are relatively few.

**Goal:**

Systematically review the effect of a portable rehabilitation robot (PRR) on the rehabilitation effectiveness of paralysed upper limbs compared with non-robotic therapy.

**Methods:**

A meta-analysis was conducted on literature that included the Fugl-Meyer Assessment (FMA) obtained from the PubMed and Web of Science (WoS) electronic databases until June 2023.

**Results:**

A total of 9 studies, which included RCTs, were completed and a meta-analysis was conducted on 8 of them. The analysis involved 295 patients. The influence on upper-limb function before and after treatment in a clinical environment is analysed by comparing the experimental group using the portable upper-limb rehabilitation robot with the control group using conventional therapy. The result shows that portable robots prove to be effective (FMA: SMD = 0.696, 95% = 0.099 to.293, *p* < 0.05).

**Discussion:**

Both robot-assisted and conventional rehabilitation effects are comparable. In some studies, PRR performs better than conventional rehabilitation, but conventional treatments are still irreplaceable. Smaller size with better portability has its advantages, and portable upper-limb rehabilitation robots are feasible in clinical rehabilitation.

**Conclusion:**

Although portable upper-limb rehabilitation robots are clinically beneficial, few studies have focused on portability. Further research should focus on modular design so that rehabilitation robots can be decomposed, which benefits remote rehabilitation and household applications.

## Introduction

The World Health Organisation (WHO) identifies stroke as a major cause of physical disability globally and ranks it as the third leading cause of death [1]. The WHO’s definition of a stroke encompasses ‘rapidly developing clinical symptoms and/or signs of focal, and at times global, disturbance of cerebral function, lasting more than 24 h or leading to death, with no apparent cause other than that of vascular origin’ [2]. The impact of stroke on daily life is profound, with approximately 75% of stroke survivors experiencing permanent disabilities. Such physical disabilities significantly diminish the quality of life. Given that the upper limbs are among the body’s most functional parts, prioritising their treatment is critical for improving overall patient outcomes and enhancing quality of life post-stroke.

Most of the current conventional rehabilitation treatments rely on the patient’s remaining motor ability, so it is often difficult to achieve the effect of comprehensive rehabilitation. In some clinical cases, patients with upper-limb dysfunction can restore their strength through conventional rehabilitation treatment, but many still fail to recover their movement level after treatment [3–6].

Studies have shown that rehabilitation after a stroke can reduce physical impairment and disability [5]. During the golden treatment period, which is 3 to 6 months after a stroke, movement can be significantly improved through a large number of repetitive tasks designed to help stroke patients take care of themselves and improve their quality of life [7]. However, several factors may contribute to inadequate or limited rehabilitation, such as insufficient allocation of medical resources, poor insurance coverage, and a lack of understanding of rehabilitation value. For example, patients in remote areas have less access to medical services than in urban areas. These problems will limit the duration and frequency of treatment for stroke patients; however, this is especially important during the golden period of rehabilitation [8].

In recent years, many research institutes have invested in the research and development of rehabilitation robots. Robotic devices used in rehabilitation may enhance arm functionality, particularly for chronic stroke patients [9,10]. To aid in the recovery of stroke survivors, a range of upper limb rehabilitation robots have been developed, showing promise in the rehabilitation sector. These robots are categorised based on their design and function, differentiating between soft and rigid robots, as well as those with single degree of freedom (DOF) and multiple DOF [11]. Each robot type offers unique benefits. The combination of robot flexibility and rigidity, along with the variations in degrees of freedom, enhances the versatility of these technologies. In the field of upper limb rehabilitation, robots are mainly classified into three types: end-effectors, exoskeleton robots, and orthoses designed for rehabilitation purposes. An end-effector is described as something that ‘Contacts a subject’s limb only at its most distal part. It simplifies the structure of the device. However, it may complicate the control of the limb position in cases with multiple possible degrees of freedom’ [11].

Rehabilitation robots can be further divided into exoskeletons and orthoses [12,13]. In general, both exoskeletons and orthoses refer to external devices worn on the body to provide support, assistance, or rehabilitation, but they have distinct characteristics. Exoskeletons typically refer to wearable robotic devices that enhance or augment the user’s physical abilities. They often incorporate powered actuators, sensors, and control systems to assist with movement or provide additional strength. Exoskeletons can be powered (active) or passive, depending on their specific design. Orthoses refer to external devices used to support and correct body structures or functions. They are typically passive and do not include active power components. Orthoses can be used for posture correction, joint stabilisation, and other purposes to improve function or alleviate symptoms [12,13]. Exoskeleton devices are defined as ‘A device with a mechanical structure that mirrors the skeletal structure of the limb, i.e. each segment of the limb associated with a joint movement is attached to the corresponding segment of the device. This design allows independent, concurrent and precise control of movements in a few limb joints’ [11]. With the development of science and technology, some orthopaedic based robots are used for upper limb rehabilitation [14].

Soft robotic wearable devices have promising potential to improve arm function and enhance the quality of life for individuals with amyotrophic lateral sclerosis (ALS) and upper limb injuries. Studies conducted by Proietti et al. [15] and Georgarakis et al. [16] demonstrate the feasibility of using portable devices for rehabilitation. Preliminary results of a study by Noronha et al. [17] also show promising prospects in using flexible wearable devices for upper limb rehabilitation in stroke patients.

Robots’ mechanical characteristics can satisfy many repetitive movements required for rehabilitation and adjust the appropriate dose through computation based on the patient’s objectively documented rehabilitation status. The clinical acceptance of robotic rehabilitation equipment is related to its ability to provide added value, which is difficult to achieve in conventional rehabilitation [6]. However, although many rehabilitation robots have been developed [6], rehabilitation robots are often expensive and bulky, which reduces the acceptance of rehabilitation robots in remote areas or small medical institutions [18]. These are the reasons that limit access to rehabilitation training for stroke patients.

Moreover, the majority of existing research seldom considers the portability of robots when assessing the effectiveness of robot-assisted therapy [19]. In this study, ‘portable’ is a relative term without clear metrics for definition. Despite various claims of developing portable rehabilitation robots, there is no universally accepted standard for defining a robot’s portability [20]. With the goal of facilitating at-home rehabilitation, portability is primarily seen in terms of the robot’s ease of transport to a patient’s home. Common sense dictates that a robot’s portability is enhanced by a reduction in its weight and volume. Nonetheless, given the differences in functionalities among robots, this study narrows the definition of ‘portable’ to weights and volumes that an average healthy adult can feasibly transport. Thus, for the purposes of this research, a ‘portable’ robot is one with dimensions and weight that are manageable by an average adult.

Based on the above statement, this study will summarise the development status of a portable rehabilitation robot (PRR) through systematic literature review, and it uses a meta-analysis method to analyse the randomised controlled trials (RCTs) [21] of PRR as an experimental group (EG) and conventional treatment as a control group (CG) to understand the effectiveness of PRR.

## Method

The literature review in this research follows the Preferred Reporting Items for Systematic Reviews and Meta-Analysis (PRISMA) guidelines for literature search, study selection, and data extraction [22].

### Literature search

Articles were systematically retrieved from the Web of Science (WoS) and PubMed electronic repositories for studies published until June 2023. Retrieval keywords are upper limb, rehabilitation, and robot; Only RCTs with stroke patients are considered research subjects, and desktop rehabilitation devices are considered EGs.

### Inclusion and exclusion criteria

Inclusion criteria: (1) articles published in English; (2) based on robotic rehabilitation, including but not limited to exoskeleton rehabilitation devices, end-effector rehabilitation devices, hand rehabilitation, wrist rehabilitation, and shoulder rehabilitation; (3) portable device, the volume and weight that healthy adults can afford, and can be operated on the table; and (4) upper limb rehabilitation robot RCT for stroke.

Exclusion criteria: (1) studies not published in full; (2) studies not targeting stroke patients; (3) studies that did not use scale methods to assess rehabilitation outcomes; (4) studies that did not describe rehabilitation devices or insufficient descriptive information; (5) review articles; (6) case reports; and (7) the source of the article is not from a peer-reviewed journal.

### Data extraction

This study utilised a combined keyword search in the Web of Science (WoS) and PubMed databases, with the search parameters being ‘Topic: (upper limb AND rehabilitation AND robot), language: (English), Document type: (Article).’ Articles were selected based on the relevance of titles and abstracts through reading, where repeated articles in different databases were filtered. Then, studies that met the inclusion criteria were reviewed one by one to finalise our list of selections. The Endnote reference manager was used to filter articles without abstracts and literature reviews. Finally, the extracted data for the meta-analyses were carefully examined.

### Literature quality assessment

This study assessed the quality of the included articles through the Physiotherapy Evidence Database (PEDro) scale (Table 1). PEDro is mainly used in clinical research to strengthen the effectiveness of physical therapy services and can be used to evaluate the quality of clinical trials [23]. There are 11 items in PEDro; each ‘YES’ item scored 1 point, whereas ‘NO’ scored 0 points. Article quality ratings are points 0–4 for low quality, 4–5 for average quality, 5–8 for good quality, and 9–10 for excellent quality. It is important to note that the first item on the PEDro scale is not included in the calculation of the total PEDro score [23].

**Table 1.** PEDro scale [23].

1. Eligibility criteria were specified	☐no	☐yes	☐where
2. Subjects were randomly allocated to groups (in a crossover study, subjects were randomly allocated an order in which treatments were received)	☐no	☐yes	☐where
3. Allocation was concealed	☐no	☐yes	☐where
4. The groups were similar at baseline regarding the most important prognostic indicators	☐no	☐yes	☐where
5. There was blinding of all subjects	☐no	☐yes	☐where
6. There was blinding of all therapists who administered the therapy	☐no	☐yes	☐where
7. There was blinding of all assessors who measured at least one key outcome	☐no	☐yes	☐where
8. Measures of at least one key outcome were obtained from more than 85% of the subjects initially allocated to groups	☐no	☐yes	☐where
9. All subjects for whom outcome measures were available received the treatment or control condition as allocated or, where this was not the case, data for at least one key outcome was analysed by ‘intention to treat’	☐no	☐yes	☐where
10. The results of between-group statistical comparisons are reported for at least one key outcome	☐no	☐yes	☐where
11. The study provides both point measures and measures of variability for at least one key outcome	☐no	☐yes	☐where

### Statistical analysis

This study uses Stata© SE 15 software for statistical analysis. Meta-analysis was used to compare the effects of PRR and conventional treatment on upper limb function before and after treatment. Because some data were not provided directly in the article, the researchers needed to estimate the information from known data, which are unified into mean and standard deviation for comparison. If the data were a median and in the interquartile range, researchers used the Quantile Estimation method proposed by McGrath for data transformation [24].

The researchers adopted an indirect comparison because it is impossible to directly compare the effects of intervention and control measures on physical body functionality. The researchers believe that it is meaningful to include a meta-analysis of five or more RCTs. After selecting, only the number of Fugl-Meyer Assessment (FMA) studies met the requirements. The EG and CG showed continuous results across different measurement scales, so standardised mean differences and 95% confidence intervals (CIs) were used to express the degree of improvement. The Chi-squared test and I2 statistics were used to determine heterogeneity, and the study adopts a random-effects Model. As for all analyses, the statistical significance criterion was set at *p* < 0.05, and statistical results were presented in forest plots.

If necessary, the original authors will be contacted to provide experimental data or publicly available data on the rehabilitation robots within the permitted scope. If it is not possible to reach the authors, an explanation will be provided.

## Results

### Research selection

**Figure 1.**
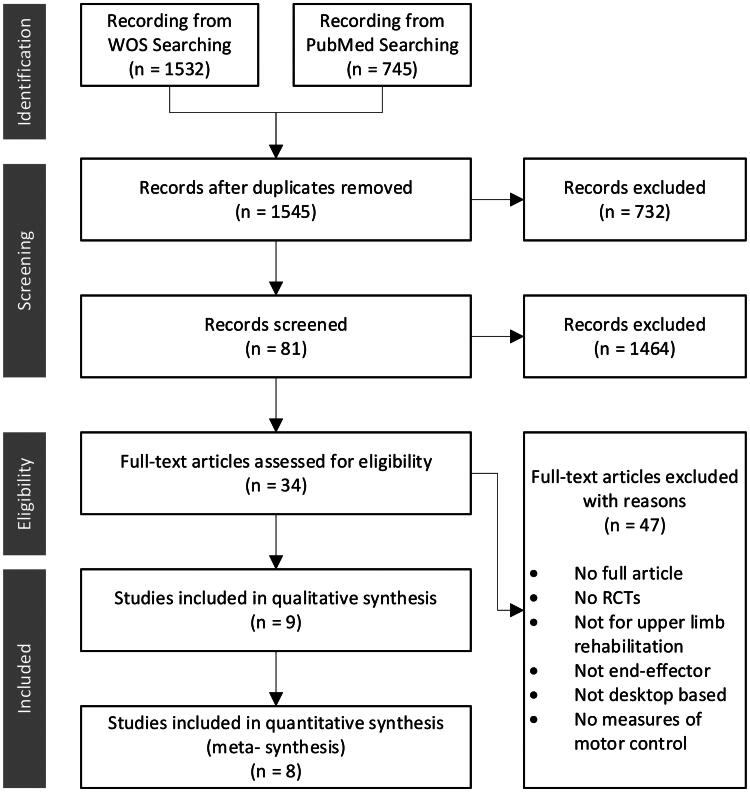
Information selection flow diagram of different phases.

Based on our search strategy, a total of 2277 articles were retrieved. After eliminating 732 duplicates, 1545 articles remained, and the remaining articles were filtered for eligibility. A total of 1464 articles were excluded by title and abstract relevancy, and 81 were acquired. Finally, through full-text reading, 9 studies were included for systematic review, and 8 were included for meta-analysis (Figure 1).

### Quality of included studies

Included studies had PEDro scores ranging from 6 to 10, with 3 studies rated 10, 3 studies rated 8, 2 studies rated 6, and 1study rated 9. The average PEDro score of the included studies was 8.33, and the experimental quality was good. Scores are shown in Table 2.

**Table 2.** PEDro Scores of included studies.

Study	2	3	4	5	6	7	8	9	10	11	Score
Hsieh et al. [25]	1	1	1	0	0	1	1	1	1	1	8
Liao et al. [26]	1	1	1	0	0	1	1	1	1	1	8
Wu et al. [27]	1	1	1	1	1	1	1	1	1	1	10
Wolf et al. [28]	1	1	1	0	0	0	1	0	1	1	6
Qian et al. [29]	1	0	1	0	0	0	1	1	1	1	6
Villafañe et al. [14]	1	1	1	1	1	1	1	1	1	1	10
Budhota et al. [30]	1	1	1	1	1	1	1	1	1	1	10
Guo et al. [31]	1	0	1	1	0	1	1	1	1	1	8
Coskunsu et al. [32]	1	1	1	1	0	1	1	1	1	1	9

### Data extraction

Table 3 represents the basic information of the experimental data extracted from the included studies, including the intervention measures, number of subjects, intervention length, at the hospital/home, tools of measurement, and results, in addition to authors and publication years. In all of the included studies, the interventions included the EG, which included both conventional and robotic treatments, and the CG, which only received conventional therapies. The number of subjects included in the study contained a total of 295 people (EG, *n* = 150; CG, *n* = 145). Hsieh [25] had the smallest number of subjects (EG, *n* = 6; CG, *n* = 6), and Wolf [28] had the most significant number of admissions, including 92 people (EG, *n* = 47; CG, *n* = 45). Only the patients in the study conducted by Wolf et al. [28] were in the subacute phase, while all the patients in the other studies had a stroke duration of over 6 months.

**Table 3.** Basic information about included studies.

Study	Interventions EG vs CG	Participations	Age(years) mean ± SD or median (IQR)	Months after stroke Mean ± SD or Median (IQR)	Place	Intervention time setting			Measuring instrument	Results
						Session duration (minutes)	Frequency (weeks)	Duration (weeks)		
Hsieh et al. [25]	RT + CT vs CT	EG: 6 CG: 6	EG: 56.04 ± 13.74 CG: 54.00 ± 8.05	EG: 21.33 ± 7.17 CG: ±	Hospital	90	5	4	FMA, MMSE, MAS, MRC	Most patients showed improvements in primary outcomes after the interventions.
Liao et al. [26]	RT + CT vs CT	EG: 10 CG:10	EG: 55.51 ± 11.17 CG: 54.56 ± 8.20	EG: 23.90 ± 13.39 CG: 22.20 ± 17.47	Hospital	90	5	4	FMA, FIM, MAL, ABILHAND	A significant difference was found between the two groups for the primary outcome measure.
Wu et al. [27]	RT + CT vs CT	EG: 14 CG: 14	EG: 55.13 ± 12.72 CG: 51.30 ± 6.23	EG: 18.00 ± 8.65 CG: 17.57 ± 9.80	Hospital	90	5	4	FMA, MAL, SIS, ADL	Large and significant effects were found in the kinematic variables, distal part of upper-limb motor impairment, and specific aspects of quality of life in favour of RT. RT may improve shoulder flexion and quality of life.
Wolf et al. [28]	RT + CT vs CT	EG: 47 CG: 45	EG: 59.1 ± 14.1 CG: 54.7 ± 12.2	EG: 3.85 ± 1.77 CG: 4.23 ± 1.54	Home	180	5	8	ARAT, WMFT, FMA	Both groups demonstrated improvement across all UE outcomes.
Qian et al. [29]	RT + CT vs CT	EG: 14 CG: 10	EG: 54.6 ± 11.3 CG: 64.6 ± 3.43	EG: 25-148 CG: 14-142	Hospital	40	5	4	FMA, ARAT, FIM, MAS	This study indicated that in the early stage after stroke, motor function in the paretic upper limbs of stroke participants could be significantly improved through traditional rehabilitation treatment and upper limb training by the NMES-robotic system.
Villafañe et al. [14]	RT + CT vs CT	EG: 16 CG: 16	EG: 67 ± 11 CG: 70 ± 12	EG: No-Data CG: No-Data	Hospital	90	3	3	NHSS, MAS, BI, MI, QuickDASH, VAS	The experimental group had a more significant reduction in pain compared with the control group at the end of the intervention.
Budhota et al. [30]	RT + CT vs CT	EG: 22 CG: 22	EG: 56.32 ± 10.37 CG: 54.59 ± 10.92	EG: 15.27 ± 15.04 CG: 13 ± 10.92	Hospital	90	3	6	FMA, ARAT, GS	Time-matched combinatory training incorporating H-Man RAT produced similar outcomes to conventional therapy alone.
Guo et al. [31]	RT + CT vs CT	EG: 10 CG: 10	EG: 56.9 ± 6.1 CG: 53.5 ± 8.3	EG: 11.7 ± 5.4 CG: 10.9 ± 7.9	Hospital	60	5	2	FMA-UL, WMFT, MAS	The upper limb function improved significantly in the experimental group
Coskunsu et al. [32]	RT + CT vs CT	EG: 11 CG: 12	EG: 59.9 ± 14.2 CG: 70.0 ± 14.0	EG: No-Data CG: No-Data	Hospital	60	5	3	FMA, ARAT, MAL	This preliminary study observed improvements in motor functions, daily living activities, and force in both groups. However, incorporating EMG-driven robotic treatment into the neurophysiological rehabilitation program did not yield additional benefits to the clinical outcomes over three weeks in acute stroke patients.

**Abbreviations:** ABILHAND, An interview-based assessment of a patient-reported measure of the perceived difficulty in using their hand to perform manual activities in daily activities; ADL, activities of daily living; ARAT, action research arm test; CT, conventional therapy; FMA, Fugl-Meyer Assessment; FMA-UL, Fugl-Meyer Motor Assessment of Upper Limb; FIM, Functional Independence Measure; GS, grip strength.; MAL, Motor Activity Log; MAS, Modified Ashworth Scale; MMSE, mini-mental state examination; MRC, Medical Research Council; NIHSS, National Institutes of Health Stroke Scale; BI, Barthel Index; MI, Motricity Index; QuickDASH, short version of the Disabilities of the Arm, Shoulder and Hand; SIS, Stroke Impact Scale; MdAS, Modified Ashworth Scale.; WMFT, Wolf Motor Function Test.; RT, robot-assisted therapy; VAS, visual analogue scale.

*Note:* a. Continuous data are expressed as mean (standard deviation), and categoric data are expressed as a number; b. *P* values are associated with the χ2 test for categoric variables and 1-way analysis of variance for continuous variables.

Regarding the statistical data on the age of the participants, all subjects were above 18 years old, and the highest average age of the EG and CG was Villafañe [14] (EG: 67 ± 11; CG: 70 ± 12), whereas Wu [27] had the lowest average age (EG: 55.13 ± 12.72; CG: 51.30 ± 6.23). The stroke severity of subjects ranged from the acute to the chronic phase. Except for Wolf [28], whose average stroke duration was less than 6 months, all other studies were above 6 months.

For intervention design, the average duration of each intervention in the included studies was 87.78 min, of which five studies were set for 90 min, the longest intervention was 180 min by Wolf [28], and the duration of interventions of all studies included conventional treatment. The average number of training sessions per week is 4.56, with the majority concentrated at 5 times per week. The average training period was 4.22 weeks. Only Wolf’ [28] was specifically designed for the home environment.

Table 4 shows information about the rehabilitation system, including system name, degree of freedom (DOF), weight, supported movements, actuators, type, and mode of wear/use. Three of the included studies used Bi-Manu-Track as the rehabilitation device for EGs. This device is mainly used for forearm and wrist training, including 1 DOF. Two studies used hand rehabilitation only, in which Villafañe [14] used the Gloreha system, which contains 6 degrees of freedom and can treat each finger and wrist. The heaviest system is Bi-Manu-Track, which weighs 46.6 kg, whereas the lightest is H-man, with 15 kg. Five of the included studies did not provide the weight of the rehabilitation system. Two sets of motors primarily drive the power source of these systems, and others such as the Hand Mentor Pro (HMP) system by Wolf [28] used a self-made McKibben air muscle as a power source. Most device types are end-effector based, whereas the neuromuscular electrical stimulation and rehabilitation robots hybrid system (NMES-Robotic system) of Qian et al. [29] is exoskeleton-based, and the Gloreha system of Villafañe [14] is orthosis.

**Table 4.** Information about the rehabilitation device.

Study	System name	DOF	Weight	Supported movements	Actuators	Type	Mode of wear/use
Hsieh et al. [25]	Bi-Manu-Track	1	46.4 kg	FA and WR	DC motor*2	End-effector based	The patient’s arm is secured within the groove of the rehabilitation machine, ensuring proper placement.
Liao et al. [26]	Bi-Manu-Track	1	46.4 kg	FA and WR	DC motor*2	End-effector based	The patient’s arm is secured within the groove of the rehabilitation machine, ensuring proper placement.
Wu et al. [27]	Bi-Manu-Track	1	46.4 kg	FA and WR	DC motor*2	End-effector based	The patient’s arm is secured within the groove of the rehabilitation machine, ensuring proper placement.
Wolf et al. [28]	Hand Mentor Pro	1	No Data	HD	McKibben air muscle	End-effector based	The device is designed to be worn on the patient’s hand and arm.
Qian et al. [29]	NMES-Robotic system	2	No Data	EL, FA and WR	Servo motor*2	Exoskeleton based	Wearable
Villafañe et al. [14]	Gloreha	6	No Data	HD	No Data	Orthosis	The device is designed to be worn on the patient’s hand and arm.
Budhota et al. [30]	H-man	2	15kg	SH, EL, FA and WR	No	End-effector based (Planar)	Wearable
Guo et al. [31]	Soft Robotic Glove	6	No Data	HD	Exclusive valve	Orthosis	The device is designed to be worn on the patient’s hand and arm.
Coskunsu et al. [32]	Hand of Hope	6	No Data	HD	motor	Exoskeleton based	The device is worn on the patient’s hand and arm

SH: shoulder, EL: elbow, FA: forearm, WR: wrist and HD: hand.

### Meta-analysis

FMA is a clinical instrument to assess the upper-extremity motor function of stroke patients. Hence, we performed a meta-analysis on FMA measurements from the selected 8 studies [25–32].

**Figure 2.**
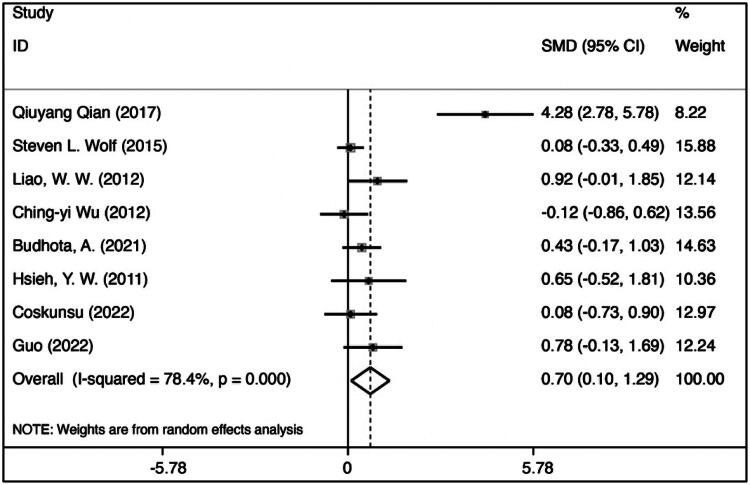
Forest plot of experimental group FMA: after vs before.

Figure 2 shows the effect size of FMA after EG intervention compared to before intervention (EG after vs EG before: SMD = 0.696, 95% = 0.099 to 1.293, *p* = 0.022; *p* < 0.05 is statistically significant), which proves that PRR is effective in upper-limb rehabilitation. Qian’s study has a more significant error rate among the included studies. This may be because the number of patients in this study is relatively small, but the length of treatment was the shortest of all of the included studies.

### Publication bias

**Figure 3.**
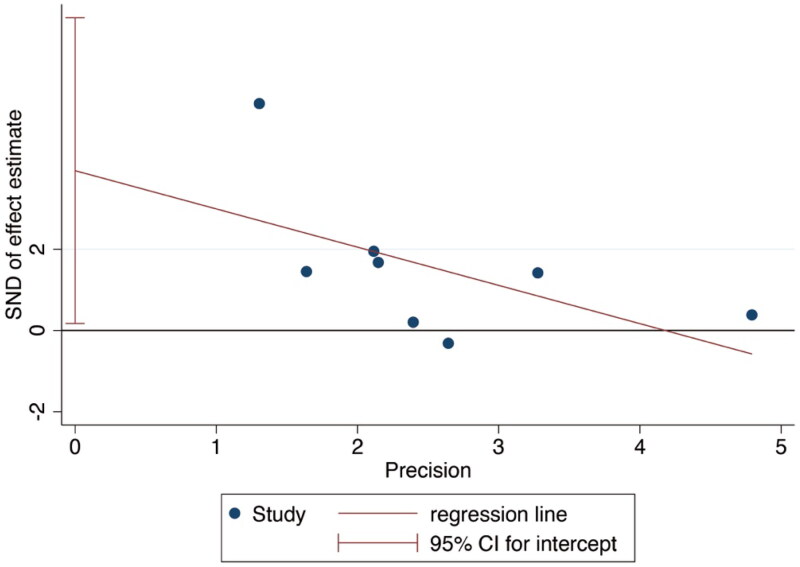
Egger’s test for publication bias plot.

Publication bias will lead to a greater possibility of publication of statistically significant research results, thus leading to bias in meta-analysis [33]. To avoid this phenomenon, we conducted bias detection for each included study. In this paper, we used the Egger’s test for bias detection, with a rate of *p* = 0.052 > 0.05 and 95% CI ranging from −0.48 to 7.68 (Table 5). Hence, the included studies are clear of publication bias (Figure 3).

**Table 5.** Egger’s test.

Std_E	Coef.	Std. Err.	*t*	*P*>|*t*|	[95% Conf. Interval]
Slope	−.9117794	.5764753	−1.58	0.165	−2.322364 .4988049
Bias	3.816237	1.579249	2.42	0.052	−.0480464 7.68052

## Discussion

Of all the included studies, three used Bi-Manu-Track [25–27], a rehabilitation device consisting of 1 DOF, and a pair of motor-driven grips, using bilateral arm training (BAT) therapy to treat the wrist [34]. Bi-Manu-Track offers three controlled modes: (1) passive-passive mode, where the robot controls both arms; (2) passive-active mode, in the unaffected arm, which drives the affected arm; and (3) active-active mode, where the affected arm actively resists the initial resistance movement, and then both arms cooperate to achieve the movement. These studies used the same dose of 90 min daily, 5 days a week, and 4 weeks of robotic training with physical therapy. However, the included studies did not expressly state the reason for this dose, and the optimal intensity remains unknown. However, regarding results, this dose appears to be effective and acceptable as long as the patient is not harmed. The literature also mentioned the impact on daily function; although robotic therapy may not be sufficient in improving daily function, the supplement of functional training can be used to learn skills necessary for daily life in order to bridge the gap between robotic training and everyday function.

Two studies focused on the hand: one of the essential limbs in the upper-limb parts. The hand has a complex musculoskeletal structure and can perform precise motor functions. Wolf et al. [28] used the HMP system, an end-effector-based rehabilitation device. HMP uses a pneumatic artificial muscle to train the movement of fingers and has a computer game that cooperates with the device to promote user experience. This research focuses more on telemedicine research carried out at home. The data pertaining to the use of robotic devices were transmitted through the internet and telephone interviews with therapists so that patients and therapists could have a more comprehensive understanding of the treatment’s effectiveness. This study also enhances the possibility of introducing robotic therapy into remote areas. In another hand rehabilitation study, Villafañe et al. [14] used orthosis; in addition, they utilised a gloved-shaped rehabilitation robot named Gloreha. The robot transmits the power of an external drive to the glove through a flexible rope, which is different from other hand rehabilitation robots. This design does not apply the device’s weight on the patient’s hand, whereas the flexible rope also offers the safety of mechanical operation. However, this device has some disadvantages. Firstly, this device is difficult to equip and requires at least 10 min of wearing time, and treatment will not be possible during this time. Secondly, the gloves require cleaning. Device weights are not available for either study, but in terms of mechanical structure, hand rehabilitation devices will likely be the lightest in this study.

Qian et al. [35] used an exoskeleton-based rehabilitation device, which contains a neuromuscular electrical stimulation and rehabilitation robot’s hybrid system (NMES-robotic system). The NMES-robotic system has relatively light mechanical properties compared to other exoskeleton-based devices. It can be worn on the patient’s shoulder and elbow. However, it still requires a metal suspension frame to hang the patient’s arm. The study shows significant efficacy, but its strict focus on early strokes makes it difficult to rule out the influence of a spontaneous recovery process.

Budhota et al. [30] used a rehabilitation robot named H-man, an unpowered table-top rehabilitation device driven by a rope with a 2D movable handlebar. H-man’s lightweight mechanical structure provides lighter weight and better portability. It is equipped with a set of visual rehabilitation software. Usability testing was conducted to analyse the ease of use of H-man and the visual effects of the software. The result showed that greater than 70% of users were satisfied with the device.

Guo et al. [31] developed a hand rehabilitation system featuring soft robotic gloves composed of retractable joint actuators and rigid components resembling bones, with a dedicated joint actuator for each interphalangeal and metacarpophalangeal joint. The system’s control unit directs the movement of the glove based on specific control commands. Actuation is achieved through the inflation and deflation of an actuator in each hand, driven by a set of miniature air pumps. The airflow within the glove is regulated by a specialised valve, ensuring that the bending and extension movements of both hands can occur independently. This valve’s operation is controlled by a relay, which opens or closes it upon receiving a command, facilitating precise control over the glove’s movements.

### Place and effect of rehabilitation

Our analysis highlights the effectiveness of portable upper limb rehabilitation, particularly when interventions are initiated after a six-month post-stroke period. This finding is consistent with the general conclusions drawn in most meta-analyses focusing on upper limb rehabilitation robots. Among the eight studies included in our analysis, only the study by Wolf et al. [28] was designed with home-based rehabilitation in mind, while the others primarily focused on the hospital setting.

Safety and compliance are crucial considerations in the development of medical equipment [36]. User behaviour, prolonged usage periods, and the variability of home environments can significantly affect the safety and effectiveness of rehabilitation interventions. Although no adverse events were reported in the studies we analysed, extending these interventions to the home setting requires additional research to affirm their safety. Current studies offer valuable insights into rehabilitation outcomes, but a deeper investigation into usability and safety is essential for a comprehensive understanding of these interventions in real-world, home-based contexts.

### Rehabilitation of upper limbs for stroke patients

Numerous therapies have been developed for post-stroke upper limb injury clinical treatment, with constraint-induced movement therapy (CIMT) being the most studied intervention in stroke patients over the past few decades [37]. The initial CIMT had the following two exercise points: (1) strengthening paralysed upper-limb exercises, specific training to enhance the affected limb, up to 6 h per day for 2 weeks; and (2) restraining the non-paralysed upper limb with gloves to facilitate the use of the more impaired limb during 90% of waking hours [38]. However, due to harsh training conditions, related research has proposed modified versions, including the primary form of the original CIMT. These modifications adopt a distributed training approach, such as reducing training time to a single day and reducing the time of restraining non-paralysed upper limbs, but it will result in extending the overall training duration. The modified CIMT treatment time varies from 30 min to 6 h per day, 2 to 7 times a week, and lasts 2 to 12 weeks [37,38].

Although CIMT has a certain degree of curative effect, many daily activities require the participation of both hands. Therefore, some related studies support BAT, which uses interlimb coordination between the affected and unaffected sides to stimulate motor interactions between the limbs [39]. BAT is also considered a viable option for post-stroke rehabilitation and is used in the Bi-Manu-Track system included in this study [25–27]. Although relevant studies have suggested that BAT is effective, the therapeutic effects of different training times and training intensities and the most practical combination of BAT and adjuvant regimens [40] are yet to be studied.

Furthermore, although these studies have assessed the rehabilitation outcomes, none of the included studies provided explanations regarding the usability and safety of the systems. In fact, the safety and compliance of the devices are crucial aspects that need to be carefully considered in the development of medical equipment [36].

### Easy to use

In the studies reviewed, the design of the robots prioritizes ease of wearability and simplicity in operation, enabling individuals without professional training to use them effectively. Notably, three of the studies [26–30] focus on the use of Bi-Manu-Track devices, which are intuitive in their use, requiring users to simply place their arms in designated rectangular grooves. The method for securing the arms becomes quickly apparent upon initial interaction with the device, underscoring the importance of user-friendly design in the context of home-based rehabilitation. On the other hand, the Gloreha device [14] necessitates a preparatory period of 10 min before use, a factor whose influence on patient motivation and adherence to rehabilitation protocols has not been fully explored. This issue is intricately linked with broader concerns regarding safety and usability. Therefore, the utility of portable robots in home-based rehabilitation hinges on their ease of use, operational simplicity, and verified safety and effectiveness, highlighting the potential for such technologies to transform patient care in home settings.

### Differences in robot size

In the literature screening, we discovered many RCT experiments on upper-limb rehabilitation robots, but few have the characteristics of portability. Many devices have bulky ground-based hosts or robotic arms as part of the rehabilitation system. The design of these rehabilitation devices usually aims to achieve more complex functions and have more DOFs. The movements of these rehabilitation robots may be 2D plane or 3D space to satisfy movements required in daily life. However, it is difficult to reduce the mechanical structure and electronic system. The use of mature and commercially available robotic arms as part of the rehabilitation system may provide a certain degree of reliability, but whether large and complex rehabilitation robots contribute positive results requires further verification.

Compared with large-sized rehabilitation robots, smaller-sized rehabilitation robots are usually portable. They can often be used on tables or worn on the patient’s limb. Although limited by the size of the system, their functions are usually monotonous and only designed for specific rehabilitation movements or limb sites. More miniature rehabilitation robots also tend to have lower DOFs, typically less than 2 DOFs, but the added benefit of portability may increase clinical acceptance.

### Structures of the robot

According to their structural characteristics, upper-limb rehabilitation devices are usually classified as end-effector based, exoskeleton based, and orthosis. In our included studies, end-effector based accounts for the majority, followed by orthosis. The end-effector based system drives the end of the limb to achieve rehabilitation. Hence, rehabilitated actions are relatively simple and have a simple structure; orthosis is directly worn on the joint of the affected area using external force or resistance to achieve rehabilitation. An orthosis is often used for hand or elbow rehabilitation, and, as a result, it tends to have good portability. Exoskeleton-based systems are usually complex in structure because each dynamic axis must be aligned with the anatomical axis of the limb, which is often bulky.

Regarding rehabilitation length and course setting, there is no consistent standard between our included studies on the length of robot-assisted therapy (RT). Most studies have RT interventions of 30 min, plus prior physiotherapy and occupational therapy, usually 90 min per treatment. However, the length of a single treatment, the course duration, and the appropriate dosage have not been mentioned in the included literature. The literature also showed that there is no fixed standard for the setting of doses [28]; hence, further study is needed for dose setting.

Research has found that rehabilitation for hand function often utilizes rehabilitation gloves, while for the arm, there is more diversity in the approaches used. It has been observed that when patients have limited residual function in the affected arm, it becomes necessary for the robot to fully assist in moving the affected arm. In such cases, flexible materials or devices that require the patient to exert some force may not be effective. Therefore, it is crucial to develop portable rehabilitation robots that incorporate both active and passive rehabilitation modes.

### Cost

Cost considerations significantly influence the development of upper limb rehabilitation robots. Qian and Bi’s study [41] indicates a wide price range for assistive robot products, from around $9,000 to $100,000. Lo et al.’s 2010 study suggests that the expenses associated with robot-assisted therapy may align with those of non-robotic therapy, particularly for long-term rehabilitation programs [4]. However, it’s crucial to recognize that this comparison primarily pertains to large-scale rehabilitation robots.

The high development costs of these robots, coupled with the relatively modest returns for patients and clinics, render the cost-effectiveness of robotic rehabilitation less than optimal. Nonetheless, the emergence of Portable Rehabilitation Robot (PRR) technology, characterized by smaller sizes and hence reduced costs, is anticipated to enhance the adoption of rehabilitation robots due to their lower prices and improved portability [18]. However, not all literature describes in detail the impact of using PRR in reducing costs. Therefore, it remains to be further validated whether the use of PRR offers a medical cost advantage compared to non-PRR rehabilitation.

### Limitation

The studies in review present notable limitations. Primarily, all trials conducted used robotic therapy subsequent to conventional therapy, preventing isolation and evaluating the effectiveness of robotic rehabilitation itself. Recognising the importance of developing portable upper limb rehabilitation robots to alleviate the substantial time and financial costs associated with regular hospital visits for rehabilitation, our investigation uncovers a shortfall in research specifically targeted at portable designs as the primary focus. Although this study introduces a straightforward definition of portable upper limb rehabilitation robots and adopts relatively expansive criteria, the number of studies aligning with these criteria remains modest. Consequently, the research area concerning portable upper limb rehabilitation robots requires additional in-depth exploration and more precise definitions in various aspects.

## Conclusion

Studies demonstrate that integrating PRR with standard therapy significantly improves upper-limb functional rehabilitation. The outcomes are either comparable to or exceed those achieved with conventional treatment alone. Robotic devices that can handle repetitive and intensive tasks reduce the labour intensity of therapists, so therapists can focus on delivering more personalised rehabilitation sessions, reducing the workforce and increasing productivity. During this study, we discovered many studies on RT but only few on portability. Although RT is currently primarily used in medical institutions, considering the impact of resource allocation and population structure, developing PRR will be an important research direction. If PRR is combined with network communication technology, these additional functions may improve the acceptance of rehabilitation robots and make household rehabilitation robots more popular.

## Data Availability

The datasets used and/or analysed during the current study available from the corresponding author on reasonable request.
